# Supplementary material for: A Critical View of the Application of the APEX Software (Aqueous Photochemistry of Environmentally-Occurring Xenobiotics) to Predict Photoreaction Kinetics in Surface Freshwaters
Source: Molecules. 2019 Dec 18;25(1):9. doi: 10.3390/molecules25010009 (PMC7017383; doi:10.3390/molecules25010009)
Supplement: Supplementary file 1 [file molecules-25-00009-s001.zip › 3.2.4_gcc-4.4.0.zip!/3.2.4_gcc-4.4.0/3.2.4_gcc-4.4.0/doc/octave/html/interpreter/Graphics-Object-Properties.html]

Graphics Object Properties - Untitled


Next: Managing Default Properties,
Previous: Graphics Objects,
Up: Advanced Plotting


---

#### 15.2.2 Graphics Object Properties

- Root Figure Properties- Figure Properties- Axes Properties- Line Properties- Text Properties- Image Properties- Patch Properties- Surface Properties- Searching Properties
